# A Inibição do Sistema Renina-Angiotensina e o Bloqueio Beta Adrenérgico podem ser Úteis para Atenuar a Cardiotoxicidade por Antraciclinas

**DOI:** 10.36660/abc.20230280

**Published:** 2023-06-02

**Authors:** Mario Fritsch Neves

**Affiliations:** 1 Departamento de Clínica Médica Universidade do Estado do Rio de Janeiro Rio de Janeiro RJ Brasil Departamento de Clínica Médica – Universidade do Estado do Rio de Janeiro, Rio de Janeiro, RJ – Brasil

**Keywords:** Antraciclinas/toxididade, Oncologia, Tratamento Farmacológico, Disfunção Ventricular Esquerda, Insuficiência Cardíaca

A cardiotoxicidade relacionada ao uso de antraciclinas representa uma grande preocupação na área de Oncologia, pois pode elevar a morbidade de muitos pacientes que recebem tratamento quimioterápico para diversos tipos de câncer. Essa toxicidade pode levar a uma disfunção sistólica do ventrículo esquerdo e, com isso, a uma maior incidência de insuficiência cardíaca, que pode resultar em mortalidade nos casos mais graves.

Uma melhor compreensão dos mecanismos envolvidos e uma melhor definição dos métodos de diagnóstico vêm propiciando significativos avanços para detecção desta complicação. Todavia, ainda não há consenso sobre os biomarcadores mais precisos para identificação precoce da cardiotoxicidade. Apesar disso, a dosagem de troponina a cada ciclo quimioterápico pode ser uma ferramenta válida para indicar uma intervenção imediata.^1 , 2^

As manifestações da cardiotoxicidade podem permanecer restritas a um dano celular miocárdico, detectável apenas pela dosagem de biomarcadores circulantes, ou variar entre uma disfunção ventricular silenciosa e insuficiência cardíaca clinicamente manifesta. Estratégias para evitar ou, pelo menos, atenuar a cardiotoxicidade induzida por antraciclinas são essenciais para melhorar o prognóstico dos pacientes em uso desta classe. Tratar os fatores de risco cardiovascular preexistentes,^3 - 5^ escolher análogos menos cardiotóxicos e administrar baixas doses cumulativas de antraciclina estão entre as recomendações atuais.^6 , 7^

Os antagonistas do sistema renina-angiotensina, como o inibidor da enzima conversora de angiotensina (IECA) e os bloqueadores dos receptores da angiotensina II (BRA), têm sido amplamente utilizados para prevenir esta complicação, mas a indicação ainda parece controversa. Por isso, a importância da revisão sistemática realizada por Avila et al.,^8^ que concluiu que a terapia com inibidores do sistema renina-angiotensina-aldosterona e com betabloqueadores não foi capaz de reduzir a mortalidade dos pacientes que fizeram uso de antraciclinas, mas foi associada com uma menor variação da fração de ejeção do ventrículo esquerdo e menor ocorrência de insuficiência cardíaca.^8^ Esses resultados indicam que a terapia neuro-hormonal pode ser importante para reduzir a morbidade destes pacientes e, com isso, melhorar a qualidade de vida, o que ainda precisa ser mensurado. A alta heterogeneidade entre os estudos considerados para a revisão sistemática aponta para a grande dificuldade de obter uma população mais homogênea devido a variedade de doenças neoplásicas de base e variação das doses das antraciclinas.

De fato, os achados desta revisão estão em acordo com uma recente metanálise que agregou 1.362 mulheres com câncer de mama em tratamento com antraciclinas ou trastuzumabe e concluiu que as terapias com betabloqueadores e inibidores do sistema renina-angiotensina foram associadas com efeitos benéficos para a preservação da fração de ejeção do ventrículo esquerdo na comparação com placebo.^9^

Estudos clínicos confirmam que o uso de IECA e BRA pode reduzir a incidência de disfunção ventricular esquerda em pacientes que recebem antraciclinas. Esses medicamentos exercem efeitos protetores contra danos causados por radicais livres, além de reduzir a resistência vascular periférica e a pressão arterial, o que pode melhorar o fluxo coronariano e o desempenho cardíaco. De forma interessante, um estudo clínico prévio demonstrou que a simples inibição da ação da aldosterona com a espironolactona foi capaz de proteger as funções sistólica e diastólica ventricular esquerda contra os efeitos adversos das antraciclinas. Não apenas a fração de ejeção, mas também os diâmetros sistólico e diastólico do ventrículo esquerdo foram protegidos pela espironolactona. Em adição, a espironolactona mostrou um efeito antioxidante, o que foi essencial devido ao estresse oxidativo induzido pela antraciclina.^10^

Os betabloqueadores também podem ser úteis na prevenção da cardiotoxicidade induzida por antraciclinas. Esses medicamentos têm efeitos positivos sobre o sistema cardiovascular, incluindo a redução da frequência cardíaca, a diminuição do consumo de oxigênio pelo coração e a melhora da função ventricular, mais especificamente aqueles com maior seletividade pelo receptor β1 cardíaco e com ação vasodilatadora. Além disso, os betabloqueadores podem ajudar a reduzir a atividade simpática, que pode contribuir para a cardiotoxicidade por antraciclinas.

É importante ressaltar que o uso de antagonistas do sistema renina-angiotensina e betabloqueadores na prevenção da cardiotoxicidade por antraciclinas deve ser cuidadosamente monitorado. Esses medicamentos podem ter efeitos colaterais significativos, incluindo hipotensão e bradicardia. A função renal deve ser acompanhada mais regularmente, pois se houver redução significativa da taxa de filtração glomerular, a inibição do sistema renina-angiotensina-aldosterona pode precipitar o aparecimento de hipercalemia. Além disso, como a presença de outras comorbidades não é infrequente, é necessário avaliar a possibilidade de interações medicamentosas entre esses fármacos e outros medicamentos em uso pelo paciente.

Em conclusão, o uso de antagonistas do sistema renina-angiotensina e betabloqueadores pode ser uma opção valiosa na prevenção da cardiotoxicidade por antraciclinas em pacientes submetidos a tratamento quimioterápico. No entanto, é importante que a terapia seja monitorada de forma mais intensa e que o paciente seja avaliado regularmente para detectar quaisquer efeitos colaterais ou interações medicamentosas. A prevenção da cardiotoxicidade por antraciclinas pode melhorar significativamente a qualidade de vida dos pacientes em tratamento oncológico e deve ser considerada como parte integrante da terapia global de cada indivíduo.
