# Supplementary material for: A Inibição do Sistema Renina-Angiotensina e o Bloqueio Beta Adrenérgico podem ser Úteis para Atenuar a Cardiotoxicidade por Antraciclinas
Source: Arq Bras Cardiol. 2023 Jun 2;120(5):e20230280. [Article in Portuguese] doi: 10.36660/abc.20230280 (PMC10263401; doi:10.36660/abc.20230280)
Supplement: Translation

# Translation: Renin-Angiotensin System Inhibition and Beta Blockade Adrenergic May Be Useful to Attenuate Cardiotoxicity by Anthracyclines

**Authors:** Mario Fritsch Neves

**Affiliations:** 1 Brasil Departamento de Clínica Médica – Universidade do Estado do Rio de Janeiro, Rio de Janeiro, RJ – Brasil

**Keywords:** Anthracyclines/toxicity, Oncology, Drug Therapy, Ventricular Dysfunction, Left, Heart Failure

Cardiotoxicity related to the use of anthracyclines is a major concern in oncology, as it can increase the morbidity of many patients receiving chemotherapy for various types of cancer. This toxicity can lead to left ventricular systolic dysfunction and, therefore, a higher incidence of heart failure, which can result in mortality in severe cases. A better understanding of the mechanisms involved and a better definition of diagnostic methods have provided significant advances in detecting this complication. However, there is still no consensus on the most accurate biomarkers for early identification of cardiotoxicity. Despite this, troponin measurement after each chemotherapy cycle can be a valid tool to indicate immediate intervention.^1 , 2^

Manifestations of cardiotoxicity may remain restricted to myocardial cell damage detectable only by measuring circulating biomarkers or vary between silent ventricular dysfunction and clinically manifest heart failure. Strategies to avoid or at least attenuate anthracycline-induced cardiotoxicity are essential to improve the prognosis of patients using this class. The current recommendations include treating pre-existing cardiovascular risk factors,^3 - 5^ choosing less cardiotoxic analogues, and administering low cumulative doses of anthracyclines.^6 , 7^

Antagonists of the renin-angiotensin system, such as angiotensin-converting enzyme inhibitors (ACEIs) and angiotensin II receptor blockers (ARBs), have been widely used to prevent this complication, but their indication still seems controversial. Therefore, the importance of the systematic review by Avila et al.^8^ concluded that therapy with renin-angiotensin-aldosterone system inhibitors or beta-blockers was not able to reduce the mortality of patients who used anthracyclines but was associated with a smaller variation in left ventricular ejection fraction and a lower incidence of heart failure.^8^ These results indicate that neurohormonal therapy may be important in reducing the morbidity of these patients and thus improving their quality of life, which still needs to be measured. The high heterogeneity among the studies considered for the systematic review indicates the great difficulty in obtaining a more homogeneous population due to the variety of underlying neoplastic diseases and variation in anthracycline doses.

In fact, this review’s findings agree with a recent meta-analysis that included 1362 women with breast cancer undergoing treatment with anthracyclines or trastuzumab, which concluded that therapies with beta-blockers and renin-angiotensin system inhibitors were associated with beneficial effects for preserving left ventricular ejection fraction compared to placebo.^9^

Clinical studies confirm that ACE inhibitors and ARBs can reduce the incidence of left ventricular dysfunction in patients receiving anthracyclines. These medications exert protective effects against damage caused by free radicals and reduce peripheral vascular resistance and blood pressure, which can improve coronary flow and cardiac performance. Interestingly, a previous clinical study showed that simple inhibition of aldosterone action with spironolactone was able to protect left ventricular systolic and diastolic functions against the adverse effects of anthracyclines. Not only the ejection fraction but also the systolic and diastolic diameters of the left ventricle were protected by spironolactone. In addition, spironolactone showed an antioxidant effect, which was essential due to the oxidative stress induced by anthracycline.^10^

Beta-blockers can also be useful in preventing anthracycline-induced cardiotoxicity. These medications positively affect the cardiovascular system, including reducing heart rate, decreasing oxygen consumption by the heart, and improving ventricular function, specifically those with greater selectivity for the cardiac β1 receptor and vasodilatory action. In addition, beta-blockers can help reduce sympathetic activity, contributing to anthracycline-induced cardiotoxicity.

It is important to note that the use of renin-angiotensin system inhibitors and beta-blockers in preventing anthracycline-induced cardiotoxicity should be carefully monitored. These medications can have significant side effects, including hypotension and bradycardia. Renal function should be monitored more regularly, as a significant reduction in glomerular filtration rate may precipitate the appearance of hyperkalemia with renin-angiotensin-aldosterone system inhibition. In addition, as other comorbidities are not uncommon, it is necessary to evaluate the possibility of drug interactions between these medications and other medications used by the patient.

In conclusion, using renin-angiotensin system inhibitors and beta-blockers may be a valuable option to prevent anthracycline-induced cardiotoxicity in chemotherapy patients. However, it is important that therapy be more intensively monitored and that the patient be regularly evaluated for any side effects or drug interactions. The prevention of anthracycline-induced cardiotoxicity can significantly improve the quality of life of patients undergoing cancer treatment and should be considered an integral part of each individual’s overall therapy.
